# Identification of Anti-Severe Acute Respiratory Syndrome-Related Coronavirus 2 (SARS-CoV-2) Oxysterol Derivatives In Vitro

**DOI:** 10.3390/ijms22063163

**Published:** 2021-03-19

**Authors:** Hirofumi Ohashi, Feng Wang, Frank Stappenbeck, Kana Tsuchimoto, Chisa Kobayashi, Wakana Saso, Michiyo Kataoka, Masako Yamasaki, Kouji Kuramochi, Masamichi Muramatsu, Tadaki Suzuki, Camille Sureau, Makoto Takeda, Takaji Wakita, Farhad Parhami, Koichi Watashi

**Affiliations:** 1 Department of Virology II, National Institute of Infectious Diseases, Tokyo 162-8640, Japan; hiro4@nih.go.jp (H.O.); kanatcmt@nih.go.jp (K.T.); k-chisa@nih.go.jp (C.K.); wsaso@nih.go.jp (W.S.); mayamasa@nih.go.jp (M.Y.); muramatsu@nih.go.jp (M.M.); wakita@nih.go.jp (T.W.); 2 Department of Applied Biological Sciences, Tokyo University of Science, Noda 278-8510, Japan; kuramoch@rs.tus.ac.jp; 3 MAX BioPharma, Inc., 2870 Colorado Avenue, Santa Monica, CA 90404, USA; fwang@maxbiopharma.com (F.W.); fstappenbeck@maxbiopharma.com (F.S.); 4 The Institute of Medical Science, The University of Tokyo, Tokyo 108-8639, Japan; 5 AIDS Research Center, National Institute of Infectious Diseases, Tokyo 162-8640, Japan; 6 Department of Pathology, National Institute of Infectious Diseases, Tokyo 162-8640, Japan; michiyo@nih.go.jp (M.K.); tksuzuki@nih.go.jp (T.S.); 7 Laboratoire de Virologie Moléculaire, Institut National de la Transfusion Sanguine, 75739 Paris, France; csureau@ints.fr; 8 Department of Virology III, National Institute of Infectious Diseases, Tokyo 208-0011, Japan; mtakeda@nih.go.jp; 9 Institute for Frontier Life and Medical Sciences, Kyoto University, Kyoto 606-8507, Japan; 10 MIRAI, JST, Kawaguchi Center Building, 4-1-8 Honcho, Kawaguchi City, Saitama 332-0012, Japan

**Keywords:** SARS-CoV-2, COVID-19, oxysterols, antiviral, coronavirus, double membrane vesicle, replication, pharmacokinetics

## Abstract

The development of effective antiviral drugs targeting the severe acute respiratory syndrome-related coronavirus 2 (SARS-CoV-2) is urgently needed to combat the coronavirus disease 2019 (COVID-19). We have previously studied the use of semi-synthetic derivatives of oxysterols, oxidized derivatives of cholesterol as drug candidates for the inhibition of cancer, fibrosis, and bone regeneration. In this study, we screened a panel of naturally occurring and semi-synthetic oxysterols for anti-SARS-CoV-2 activity using a cell culture infection assay. We show that the natural oxysterols, 7-ketocholesterol, 22(*R*)-hydroxycholesterol, 24(*S*)-hydroxycholesterol, and 27-hydroxycholesterol, substantially inhibited SARS-CoV-2 propagation in cultured cells. Among semi-synthetic oxysterols, Oxy210 and Oxy232 displayed more robust anti-SARS-CoV-2 activities, reducing viral replication more than 90% at 10 μM and 99% at 15 μM, respectively. When orally administered in mice, peak plasma concentrations of Oxy210 fell into a therapeutically relevant range (19 μM), based on the dose-dependent curve for antiviral activity in our cell-based assay. Mechanistic studies suggest that Oxy210 reduced replication of SARS-CoV-2 by disrupting the formation of double-membrane vesicles (DMVs); intracellular membrane compartments associated with viral replication. Our study warrants further evaluation of Oxy210 and Oxy232 as a safe and reliable oral medication, which could help protect vulnerable populations with increased risk of developing COVID-19.

## 1. Introduction

Coronavirus disease 2019 (COVID-19), caused by infection with the severe acute respiratory syndrome-related coronavirus 2 (SARS-CoV-2), has drastically impacted public health and, on a global scale, caused enormous harm to human societies and their economic vitality. In the search for effective treatments for COVID-19, understandably, the repurposing of existing FDA-approved drugs has been given high priority due to their known safety profiles [1]. For example, remdesivir (RDV), which was originally designed as an anti-ebola virus agent, has been repurposed to become the first and, to date, the only FDA-approved drug treatment for SARS-CoV-2 infection. Similarly, chloroquine (CLQ) and hydroxychloroquine, which are used to control malaria, have been investigated as COVID-19 treatments [2]. Beyond drug repurposing [3], other approaches are urgently needed to invigorate discovery research for new, specific, and potent anti-COVID-19 drugs.

Naturally occurring oxysterols include metabolites of cholesterol involved in the biosynthesis of steroid hormones, vitamin D, bile acids, and other crucial signaling molecules [4,5]. Beyond their role as passive and transient metabolites, endogenous oxysterols are increasingly recognized as lipid signaling molecules that can regulate a range of physiological processes, including lipid homeostasis, transport, and metabolism, as well as the immune response [5]. In recent years, numerous reports have ascribed broad-spectrum antiviral properties to naturally occurring oxysterols. For example, 20(*S*)-hydroxycholesterol (20(*S*)-OHC) and 22(*S*)-hydroxycholesterol (22(*S*)-OHC) reduced the infection of hepatitis B virus [6]; 25-hydroxycholesterol (25-OHC) and 27-hydroxycholesterol (27-OHC) displayed antiviral activities against herpes simplex virus [7], human papillomavirus-16, human rhinovirus [8], murine norovirus [9], rotavirus [10], and Zika virus [11].

In this study, we focused on oxysterols, including naturally occurring and semi-synthetic oxysterols, to identify potent anti-SARS-CoV-2 agents since we have already developed various semi-synthetic oxysterols as drug candidates in the context of cancer, fibrotic diseases, and bone regeneration: Oxy133, an allosteric activator of Hedgehog (Hh) signaling, was designed for orthopedic applications, such as bone repair and spine fusion [12,13,14]; Oxy186, an inhibitor of Hh signaling that acts downstream of the Smoothened (Smo) receptor, was designed as a potential anti-tumorigenic agent [15], and Oxy210 was designed for application in cancer and fibrosis through dual inhibition of Hh and transforming growth factor β (TGFβ) signaling [16]. In the present report, using cell-based analysis, we demonstrate that Oxy210 and its analog, Oxy232, display superior anti-SARS-CoV-2 activity compared to the natural oxysterols, 7-ketocholesterol (7-KC), 22(*R*)-hydroxycholesterol (22(*R*)-OHC), 24(*S*)-hydroxycholesterol (24(*S*)-OHC), and 27-OHC. Importantly, Oxy210 reduced viral replication and the formation of double-membrane vesicles (DMVs), known RNA replication factories of coronaviruses and other RNA viruses [17,18,19]. Oral administration of a single dose of Oxy210 at 200 mg/kg in mice resulted in a peak plasma concentration (C_max_) of about 19 μM, which exceeds both the 50% maximal inhibitory concentration (IC_50_) (5.5 μM) and 90% maximal inhibitory concentration (IC_90_) (8.6 μM), respectively, determined by our cell-based assay. These data provide foundational evidence for Oxy210 and Oxy232 as potential anti-COVID-19 candidates for further therapeutic development in the future.

## 2. Results

### 2.1. Natural Oxysterols Have Antiviral Activity against SARS-CoV-2 Infection

**Figure 1:**
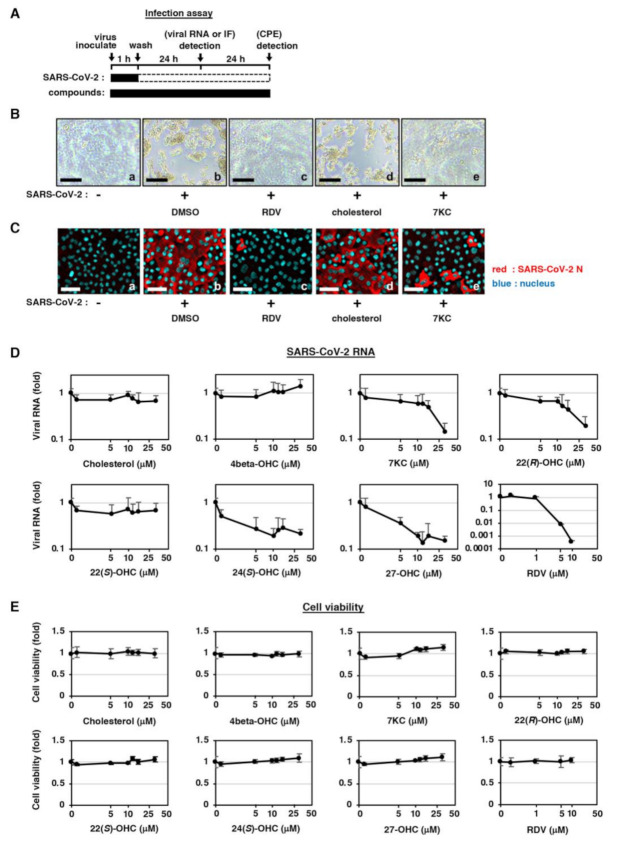
Oxysterols inhibit SARS-CoV-2 infection. (**A**) Schematic model of the SARS-CoV-2 infection assay. VeroE6 cells overexpressing transmembrane protease, serine 2 (TMPRSS2) were inoculated with SARS-CoV-2 in the presence of compounds for 1 h, followed by washing out the free virus and incubating the cells with the compounds for 24 or 48 h. Viral RNA in the culture supernatant and viral N protein in the cells was quantified at 24 h post-inoculation by real-time RT-PCR and immunofluorescence analyses, respectively. Cytopathic effects (CPE) were viewed under a microscope at 48 h post-inoculation. Solid and dashed boxes indicate the periods that the cells were treated with and without the compounds or the virus, respectively. (**B**) Images of the cells treated with the virus in the presence of dimethyl sulfoxide (DMSO), 10 μM Remdesivir (RDV), 30 μM cholesterol, or 30 μM 7-ketocholesterol (7-KC). Scale bar, 100 μm. (**C**) Viral N protein in the cells was detected by indirect immunofluorescence analysis. The red and blue signals represent viral N protein and nuclei, respectively. Scale bar, 50 μm. (**D**) Dose-response curves for SARS-CoV-2 RNA upon treatment with the compounds as indicated. OHC: hydroxycholesterol. Viral RNAs in the culture supernatant were quantified by real-time RT-PCR and plotted against compound concentrations up to 30 μM. (**E**) Viability of cells treated with compounds as indicated for 24 h was quantified using an MTT assay.

In this study, we used a cell-based SARS-CoV-2 infection system previously reported [20]. This infection system uses VeroE6 cells stably overexpressing the TMPRSS2 gene, which is a member of type II transmembrane serine proteases. Cells were treated with test compounds for 1 h during inoculation with a clinical isolate of SARS-CoV-2 at a multiplicity of infection (MOI) of 0.001, followed by washing out free virus and incubating the cells with test compounds for 24 h or 48 h (Figure 1A and Materials and Methods). SARS-CoV-2 propagation in VeroE6/TMPRSS2 cells induced a cytopathic effect (CPE) at 48 h post-virus inoculation (Figure 1B, panel b), and the treatment with remdesivir (RDV), a known replication inhibitor of SARS-CoV-2 [2], blocked the virus-induced CPE (Figure 1B, panel c). SARS-CoV-2 propagation visualized by detecting viral nucleocapsid (N) protein by immunofluorescence (IF) analysis was also blocked by RDV (Figure 1C, panels b,c, red). Using this assay, we evaluated the antiviral effect of cholesterol and 7-ketocholesterol (7-KC) as a representative of natural oxysterols. 7-KC, but not cholesterol, reduced the SARS-CoV-2-induced CPE (Figure 1B, panels d,e) and the spread of infection (Figure 1C, panels d,e). To quantify antiviral activity, we measured viral RNA production in the culture supernatant and cell viability upon treatment with natural oxysterols or cholesterol at 24 h post-inoculation. Cholesterol, 4beta-hydroxysterol (4beta-OHC) and 22(*S*)-hydroxycholesterol (22(*S*)-OHC), did not show apparent reductions in viral RNA, while 7-KC, 22(*R*)-hydroxycholesterol (22(*R*)-OHC), 24(*S*)-hydroxycholesterol (24(*S*)-OHC), and 27-hydroxysterol (27-OHC) reduced the production of viral RNA by 80%–86% as compared to control (Figure 1D and Supplementary Figure S1). Evaluation of host cell viability showed no cytotoxic effect of the test compounds up to 30 μM, which is the maximum concentration in the SARS-CoV-2 infection assay shown in Figure 1D,E. The IC_50_s, IC_90_s, and 50% maximal cytotoxic concentrations (CC_50_s) for these compounds are summarized in Table 1. These findings suggest that the oxysterols inhibited SARS-CoV-2 propagation without showing cytotoxicity.

### 2.2. Semi-Synthetic Oxysterol Derivatives, Oxy210, Oxy186, and Oxy232 Inhibit SARS-CoV-2 Production

**Figure 2:**
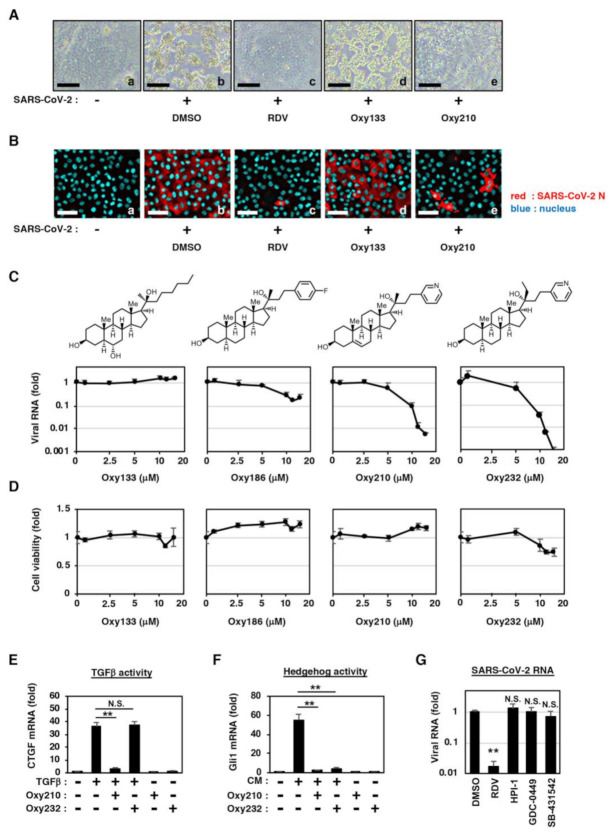
Oxy210, an oxysterol derivative, potently inhibits the SARS-CoV-2 propagation and alleviates the virus-induced CPE. (**A**) Virus-induced CPE was examined in the cells inoculated with the virus in the presence of DMSO, 10 μM RDV, 10 μM Oxy133, or 10 μM Oxy210. Scale bar, 100 μm. (**B**) Viral N protein in the cells was detected by immunofluorescence analysis as described in Figure 1C. Scale bar, 50 μm. (**C**) Dose-response curves for viral RNA upon treatment with oxysterol derivatives as indicated. The secreted viral RNA in the culture supernatant at 24 h post-inoculation was quantified by real-time RT-PCR and plotted against compound concentration. The chemical structures of oxysterols are also shown above the graphs. (**D**) Viability of cells treated with the compounds was quantified using an MTT assay. (**E**,**F**) Inhibitory effects toward transforming growth factor β (TGFβ) and hedgehog (Hh) signaling. NIH3T3 cells pretreated with or without 10 μM Oxy210 or 10 μM Oxy232 for 2 h were stimulated with 20 ng/mL TGFβ (**E**) or with conditioned medium (CM) from CAPAN-1 human pancreatic tumor cells that contain Shh (**F**) [15,16] in the presence or absence of the compounds at 10 μM. Cellular mRNAs were extracted to quantify a TGFβ-downstream gene, connective tissue growth factor (CTGF) (**E**), a Hh target gene, Gli1 (**F**), and Oaz1 for normalization of CTGF and Gli1 (**E**,**F**). (**G**) At 24 h post-inoculation, Viral RNA produced from the cells treated with DMSO, 10 μM RDV, 10 μM HPI-1, 10 μM GDC-0449, or 10 μM SB-431542, was quantified with real-time RT-PCR. All data are shown with error bars indicating S.D., ** *p* < 0.01 vs. DMSO; N.S., not significant, with Student’s *t*-test.

Although the natural oxysterols, 7-KC, 22(*R*)-OHC, 24(*S*)-OHC, and 27-OHC showed modest anti-SARS-CoV-2 activities, their physiological concentrations are far below μM ranges [21,22] in the circulation of healthy humans, suggesting their limited role, if any, in preventing viral infection under physiological conditions. In the search for oxysterols with improved antiviral activity, we evaluated the potential of semi-synthetic oxysterol derivatives for SARS-CoV-2 inhibition. SARS-CoV-2-induced CPE and virus propagation were blocked when treated with Oxy210 but not Oxy133 (Figure 2A,B, panels d and e). Quantification of SARS-CoV-2 RNA in the culture supernatant at 24 h post-inoculation also showed that Oxy210 and its structurally related derivatives, Oxy186 and Oxy232, reduced viral RNA level in a dose-dependent manner, while Oxy133 did not show antiviral activity up to 15 μM (Figure 2C). The antiviral activity of Oxy186 was almost equivalent to that of the natural oxysterols shown earlier; the maximum reduction in viral RNA was 83% when used at 12 μM as compared to control (Figure 2C, note that the viral RNA shown in logarithm scale). On the other hand, Oxy210 and Oxy232 showed much higher antiviral potencies; viral RNA production was reduced by 99.4% (Oxy210) and 99.9% (Oxy232) at the maximum at 15 μM (Figure 2C). No significant cytotoxicity by Oxy186 and Oxy210 was found up to 15 μM, the maximum concentration in the infection assay; however, Oxy232 slightly reduced cell viability when used at concentrations above 10 μM (Figure 2D). Due to the greater availability of Oxy210 we performed further studies with this oxysterol analog. The 50% and 90% maximal inhibitory concentration (IC_50_, IC_90_) and 50% maximal cytotoxic concentration (CC_50_) of Oxy210 were 5.5 μM, 8.6 μM, and >15 μM (Table 1), respectively.

We previously reported that Oxy210 inhibited Hedgehog (Hh) and transforming growth factor β (TGFβ) signaling in fibroblastic cells and tumor cells [16]. In contrast, Oxy232, a close structural analog of Oxy210, is devoid of significant TGFβ inhibitory properties (Figure 2E) but retains inhibitory activity toward Hh signaling (Figure 2F), suggesting that inhibition of TGFβ signaling is not responsible for the anti-SARS-CoV-2 activity. Consistent with this observation, treatment with the TGFβ signaling inhibitor, SB431542, did not significantly inhibit the production of viral RNA (Figure 2G). In addition, the inactivation of the Hh pathway by either HPI-1 or GDC0449 did not decrease the viral RNA levels (Figure 2G). These data suggest that Oxy210, Oxy232, and other antiviral oxysterol analogs, inhibit SARS-CoV-2 production independently of the inhibition of Hh or TGFβ signaling pathways.

### 2.3. Oxy210 Inhibits the Intracellular SARS-CoV-2 Replication and Formation of Double Membrane Vesicles

**Figure 3:**
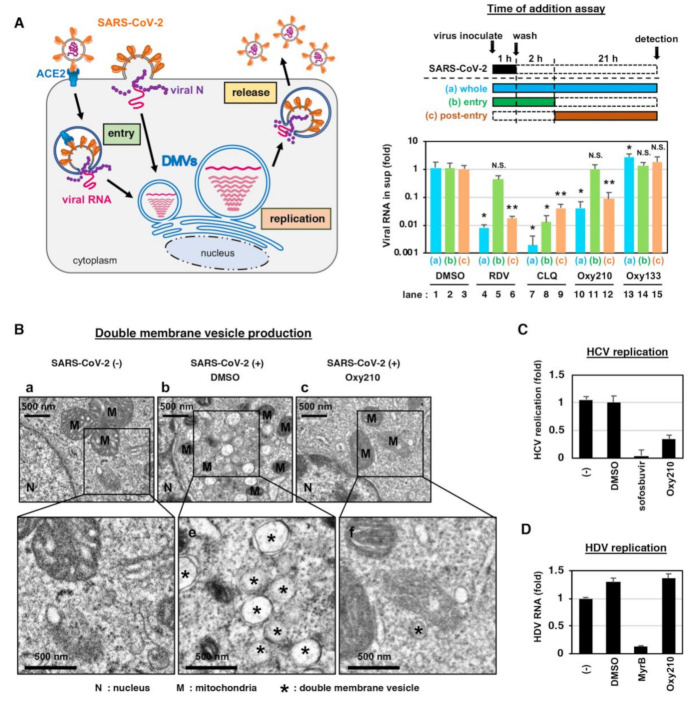
Oxy210 inhibits the SARS-CoV-2 genome replication. (**A**) Determination of the target step of compounds in the SARS-CoV-2 life cycle using time of additional analysis. The left diagram shows the life cycle of SARS-CoV-2, including the steps for viral entry, replication, and release. The upper right diagram shows the experimental procedures of the time of additional analysis. The assay was performed under three different conditions (a, whole; b, entry; c, post-entry): (a) the cells were treated with the compounds for 24 h throughout the whole procedure (whole life cycle); (b) compounds were added during the 1 h virus inoculation and then removed after an additional 2 h treatment (entry); (c) compounds were added at 2 h post-inoculation and presented for the remaining 21 h until harvest (post-entry). Solid and dashed boxes indicate the periods of presence and absence of the compounds, respectively. The lower right graph shows the real-time RT-PCR quantified viral RNA produced from the cells treated with 15 μM RDV, 15 μM CLQ, 10 μM Oxy210, or 10 μM Oxy133 under the three experimental conditions. All data are shown with error bars indicating S.D., * *p* < 0.05 vs. DMSO; ** *p* < 0.01 vs. DMSO; N.S., not significant; with Student’s *t*-test. (**B**) SARS-CoV-2 infected (panel b,c) or uninfected (panel a) cells were treated with the compounds (b, DMSO; c, 10 μM Oxy210) as indicated and examined with electron microscopy. Images in panels d–f show the insets in panels a–c, respectively, at higher magnification. N, nucleus; M, mitochondria; *, double-membrane vesicle. (**C**) Hepatitis C virus (HCV) replication was evaluated by measuring the luciferase activity in LucNeo#2 cells carrying the discistronic HCV NN (genotype-1b) subgenomic replicon RNA and the luciferase gene (see Materials and Methods), treated with or without DMSO, 10 μM Oxy210, or 1 μM sofosbuvir as a positive control for 48 h. (**D**) Hepatitis D virus (HDV) replication was measured by quantifying HDV RNA using real-time RT-PCR in HepG2-hNTCP-C4 cells infected with HDV and treated with or without DMSO or 10 μM Oxy210 for six days. 200 nM myrcludex-B (MyrB) was used as a positive control to inhibit HDV infection.

To determine which steps in the SARS-CoV-2 life cycle (Figure 3A, left) were inhibited by Oxy210, we performed a time of addition assay (Figure 3A, upper right). We examined the antiviral effect of Oxy210 in three different experimental groups, with different compound treatment times (Figure 3A, a–c); (a) Compounds were treated during the 1 h virus inoculation and the additional 23 h up to detection to represent the whole life cycle (a, blue); (b) Compounds were present during the 1 h virus inoculation and an additional 2 h, and then removed to represent the virus entry process (b, green); and (c) Compounds were added 2 h after virus inoculation and were present for the remaining 21 h to represent the post-entry period (c, orange). We confirmed that Chloroquine (CLQ), a reported SARS-CoV-2 entry inhibitor that acts through modulation of intracellular pH [2,23,24], showed the most inhibitory effect when introduced in the entry step of infection (Figure 3A, lower right, lane 8). (Because of the multiple rounds of viral re-infection in our assay, entry inhibitors can also show antiviral effects when introduced at post-entry (Figure 3A, lower right, lane 9)). We also confirmed the mode of action of RDV, a reported inhibitor of intracellular viral RNA replication [25], by showing no significant effect on the virus entry-step (Figure 3A, lower right, lane 5) and a remarkable inhibition of post-entry (Figure 3A, lower right, lane 6). In this assay system, Oxy210, but not the negative control, Oxy133, clearly reduced viral RNA levels when present during the whole life cycle and the post-entry, but not at the entry phase, similar to the effects of RDV (Figure 3A, lower right, lanes 10–12). This finding suggests that Oxy210 targets intracellular virus replication rather than viral entry.

Coronaviruses generally induce the formation of unique membrane compartments, called double-membrane vesicles (DMVs), which enable an efficient viral RNA replication [18,26]. We found that DMV formation occurs with infection by SARS-CoV-2 in VeroE6/TMPRSS2 cells (Figure 3B, panels b,e, *). Interestingly, treatment with Oxy210 remarkably reduced the DMV formation in the SARS-CoV-2-infected cells (Figure 3B, panels c,f, *). We examined the specificity of Oxy210′s effect on DMV-dependent virus replication by evaluating the antiviral effect on hepatitis C virus (HCV) and hepatitis D virus (HDV), which are other RNA viruses that drive replication in a DMV-dependent and -independent manner, respectively [26,27]. Similar to the effect of an HCV polymerase inhibitor, sofosbuvir, used as a positive control, Oxy210 reduced the DMV-dependent RNA replication of HCV (Figure 3C), while the antiviral activity was not observed in HDV infection that was inhibited by the positive control, MyrB (Figure 3D). These data are consistent with the idea that Oxy210 specifically inhibits the DMV-dependent virus replication, although it remains to be determined whether Oxy210 directly inhibits the DMV formation machinery, which we will examine in future studies (see the discussion below).

### 2.4. Pharmacokinetics of Oxy210 in Mice

**Figure 4:**
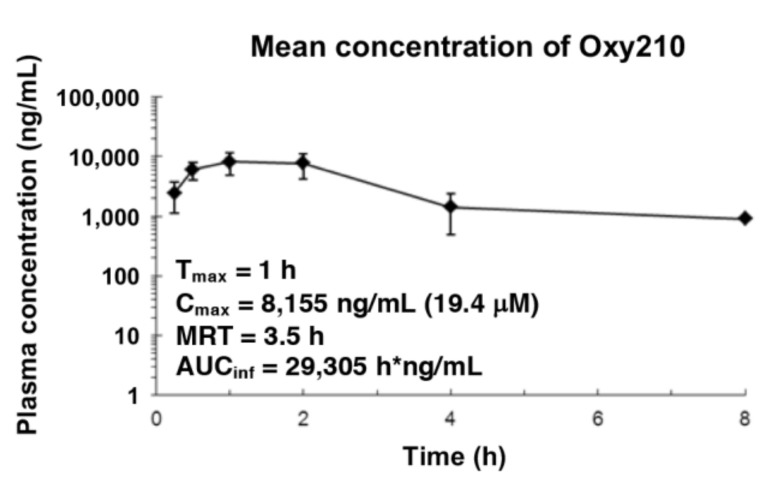
Pharmacokinetics of Oxy210 in mice. A single dose of Oxy210 at 200 mg/kg, formulated in 3% DMSO + 7% Ethanol + 5% PEG400 + 85% corn oil, was administered to balb/c mice by oral gavage. Plasma samples were taken at 0.25, 0.5, 1, 2, 4, and 8 h, followed by LC/MS analysis of the plasma to quantify Oxy210 concentrations.

Given its higher anti-SARS-CoV-2 potency compared to the natural oxysterols, we questioned whether oral administration of Oxy210 in mice would result in plasma concentrations high enough to sustain significant antiviral activity in vivo. According to a previously established protocol [16], a single dose of Oxy210 at 200 mg/kg was orally administered to mice, and the plasma concentration was examined at 0.25, 0.5, 1, 2, 4, and 8 h (h). Oxy210 was well tolerated by the mice in this experiment. After 1 h (T_max_), Oxy210 reached a peak plasma concentration (C_max_) of 8,155 ng/mL (19.4 μM) with an overall exposure of 29,305 h*ng/mL, as measured by the area under the curve (AUC) (Figure 4)

In a separate study, Oxy210 was administered to mice via a chow diet containing 4 mg Oxy210/g of food. Oxy210 plasma concentrations were measured at 24, 48, and 96 h. No adverse effects or significant loss of body weight was recorded during this 96 h experiment. Accumulation of Oxy210 in plasma was greatest after 96 h, averaging at 2682 ng/mL (6.4 μM) (Supplementary Table S1). The concentration in the liver and the lung after 96 h was higher at 6,869 ng/mL (16.3 μM) and 4,137 ng/mL (9.8 μM), respectively (Supplementary Table S1). These pharmacokinetic data indicate that oral administration of Oxy210 in mice, via oral gavage or mixed into a chow diet, results in plasma and lung concentrations that could sustain significant anti-SARS-CoV-2 activity in vivo.

## 3. Discussion

In this study, we have evaluated the anti-SARS-CoV-2 activity of a collection of naturally occurring and semi-synthetic oxysterol derivatives in cell cultures. Oxysterols are a class of understudied molecules that, until recently, have rarely been considered as a source of therapeutic drug candidates. In fact, most naturally occurring oxysterols cannot be ideal drug candidates for several different reasons, such as metabolic instability and overlapping biological activities. For example, 25-OHC, in addition to its antiviral properties, can also amplify the activation of immune cells and increases the production of potentially harmful immune mediators, which are linked to the development of atherosclerosis [5]. Semi-synthetic oxysterol derivatives, by contrast, often possess improved drug-like properties, in terms of potency, selectivity, metabolic stability, and drug safety characteristics, compared to their naturally occurring counterparts. Given the urgency of the COVID-19 pandemic, we seek to establish a suitable drug development candidate with potent anti-SARS-CoV-2 activity that does not elicit unrelated or untoward pharmacological responses. In this study, Oxy210, a semi-synthetic oxysterol, was identified as a potent replication inhibitor of the SARS-CoV-2, which reduced the formation of DMVs. The peak plasma concentration of Oxy210 reached after administration via oral gavage (19 μM) and the plasma (6.4 μM) and lung (9.8 μM) concentrations reached after administration through diet, fall into a therapeutically meaningful range as they approach or exceed the IC_50_ (5.5 μM) and IC_90_ (8.6 μM) concentrations observed in our cell-based assays. Therefore, Oxy210 and its analogs, such as Oxy232, could potentially serve as drug candidates targeting COVID-19.

We have previously characterized Oxy210 as a Hh and TGFβ signaling inhibitor [16] and have demonstrated protective effects of Oxy210 in a mouse model of idiopathic pulmonary fibrosis (IPF) (Parhami et al., unpublished observations). Oxy232, a close structural analog of Oxy210 but devoid of significant TGFβ pathway inhibitory properties, displayed anti-SARS-CoV-2 activity comparable to Oxy210, suggesting that the mechanisms of the anti-SARS-CoV-2 activity shared by Oxy210 and Oxy232 are likely unrelated to TGFβ inhibitory properties exhibited by Oxy210. This notion was further supported by the lack of antiviral activity displayed by a TGFβ pathway inhibitor, SB431542 (10 μM). Also, the antiviral activity is not likely to be due to the inhibition of the Hh pathway, as suggested by the lack of antiviral activity of Hh pathway inhibitors, HPI-1 (10 μM) and GDC0449 (10 μM). Given the absence of unrelated biological activities, such as TGFβ inhibition, Oxy232 may be a preferable drug candidate compared to Oxy210.

A recent publication reported the significant anti-SARS-CoV-2 activity of 27-OHC; low concentrations of 27-OHC inhibited post-entry, and higher concentrations inhibited the viral entry process [21]. Our time-of-addition analysis suggests that Oxy210 predominantly inhibits the post-entry process, which includes viral RNA replication in the replication factory and the following assembly of progeny virus and its secretion. We observed the formation of DMVs in SARS-CoV-2-infected cells, as previously reported [18,28]. DMVs, membrane compartments separated from the nuclease/protease-rich cytosol, are generally considered to be sites for efficient replication of genomic RNA of coronaviruses and of certain other RNA viruses, such as HCV [26]. DMVs are also very likely to be important in SARS-CoV-2 replication [17]. We showed that the production of DMVs, induced by SARS-CoV-2, was substantially reduced with Oxy210 treatment. Antiviral effects of Oxy210 were also observed during the replication of HCV, a virus that depends on DMVs for replication, but not with HDV, a virus that replicates independently of DMVs. These findings suggest that Oxy210 specifically reduces DMV-dependent virus replication. It is not clear, however, whether Oxy210 directly inhibits the formation of DMVs. Further studies will be performed in the future to analyze the mode of action for the antiviral activity of Oxy210 and its analogues, and to elucidate the molecular mechanisms underlying their inhibitory effects on SARS-CoV-2 replication and DMV formation.

It is noteworthy to outline the potential advantages of semi-synthetic oxysterols as anti-SARS-CoV-2 agents, compared to established antiviral compounds, such as RDV:

(1) RDV has to be administered intravenously, most often in a hospital setting, whereas the oxysterols could potentially be dosed orally (via a pill or liquid gel). A safe and reliable oral medication could be administered at an earlier stage, for example, at the time of confirming a SARS-CoV-2 infection, and potentially benefit asymptomatic individuals and those at increased risk of infection who have close contact with infected individuals, including medical care workers, as a prophylactic treatment.

(2) Oxysterols reprogram the host cell, interfering with the ability of the virus to use its machinery to replicate, reducing the likelihood of emerging drug resistance, and likely possess universal antiviral activity against SARS-CoV-2 mutant strains.

(3) The scaling up and manufacturing of oxysterol-based drug candidates is expected to be straightforward and process friendly, especially when compared to the manufacturing process of RDV, which is rather difficult to prepare at scale.

We conclude that semi-synthetic oxysterol derivatives, such as Oxy210 and Oxy232, could be promising leads in the search for COVID-19 drug candidates used alone or in combination with other therapies currently FDA approved or under investigation, such as RDV, convalescent plasma, or antibody treatments.

## 4. Materials and Methods

### 4.1. Compounds and the Synthesis of Oxysterol Derivatives

Commercially available oxysterols were obtained from Sigma Aldrich (St. Louis, MO, USA). Oxy133, Oxy186, and Oxy210 were prepared as previously described [12,15,16]. Oxy232 was prepared via a similar three-step synthesis described for Oxy186 and Oxy210, except for using ethyl magnesium bromide (instead of methyl magnesium bromide or methyl lithium) in step three, as explained in the Supplementary Notes of the Supplementary Materials. Other procedural details and spectral data for Oxy133, Oxy186, Oxy210, and Oxy232 are also provided (Supplementary Notes and Supplementary Figures S2–S5). RDV was purchased from Chemscene (Monmouth Junction, NJ, USA); CLQ was purchased from Tokyo Chemical Industry (Tokyo, Japan); GDC-0449 was purchased from APExBIO (Boston, MA, USA), HPI-1 and SB-431542 were purchased from Cayman Chemical (Ann Arbor, MI, USA), sofosbuvir was purchased from MedChemExpress (Monmouth Junction, NJ, USA), and Myrcludex-B was synthesized by Scrum (Tokyo, Japan).

### 4.2. Cell Culture

VeroE6/TMPRSS2 cells, VeroE6 cells overexpressing transmembrane protease, serine 2 (TMPRSS2) [20,29], were cultured in Dulbecco’s modified Eagle’s medium (DMEM; Wako, Osaka, Japan) supplemented with 10% fetal bovine serum (FBS; Sigma Aldrich, St. Louis, MO, USA), 10 units/mL penicillin, 10 μg/mL streptomycin, 10 mM HEPES (pH 7.4), and 1 mg/mL G418 (Nacalai, Kyoto, Japan) at 37 °C in 5% CO_2_. During the infection assay, 10% FBS was replaced with 2% FBS, and G418 removed. LucNeo#2 cells, carrying HCV subgenomic replicon, were kindly provided by Kunitada Shimotohno at National Center for Global Health and Medicine [30] and were cultured in DMEM supplemented with 10% FBS, 10 units/mL penicillin, 10 μg/mL streptomycin, 0.1 mM non-essential amino acids (Invitrogen, Carlsbad, CA, USA), 1 mM sodium pyruvate, 10 mM HEPES (pH 7.4), and 0.5 mg/mL G418 at 37 °C in 5% CO_2_. HepG2-hNTCP-C4 cells, a HepG2 cell clone overexpressing the HDV entry receptor, sodium taurocholate cotransporting polypeptide (NTCP), and highly susceptible to HDV infection [6] were cultured in GlutaMax (Invitrogen, Carlsbad, CA, USA) supplemented with 10 units/mL penicillin, 10 μg/mL streptomycin, 10% FBS, 10 mM HEPES (pH 7.4), 50 μM hydrocortisone, and 5 μg/mL insulin at 37 °C in 5% CO_2_.

### 4.3. SARS-CoV-2 Infection Assay

SARS-CoV-2 was handled in a biosafety level 3 (BSL3) facility. We used the SARS-CoV-2 Wk-521 strain, a clinical isolate from a COVID-19 patient, and obtained viral stocks by infecting VeroE6/TMPRSS2 cells [20]. VeroE6/TMPRSS2 cells were inoculated with SARS-CoV-2 at an MOI of 0.001 (Figure 1B,C and Figure 2A,B), 0.003 (Figure 1D, Figure 2C and Figure 3A), and 1 (Figure 3B) for 1 h and unbound virus removed by washing. Cells were cultured for 24 h prior to measuring extracellular viral RNA or detecting virally encoded N protein, for 48 h to detect cytopathic effects (CPE), and for 7 h to observe cells by electron microscopy. Compounds were added during virus inoculation (1 h) and after washing (24 or 48 h) except for the time of addition assay shown in Figure 3A.

For the time of addition assay, we added compounds with three different timings (Figure 3A): (a) present during the 1 h virus inoculation and maintained throughout the 23 h infection period (whole life cycle); (b) present during the 1 h virus inoculation and for an additional 2 h and then removed (entry); or (c) added at 2 h after virus inoculation and present for the remaining 21 h until harvest (post-entry). Inhibitors of viral replication such as remdesivir (RDV) were expected to show antiviral activity in (a) and (c), but not (b), while entry inhibitors including CLQ reduce viral RNA in all three conditions [2].

### 4.4. Quantification of Viral RNA

Viral RNA in the culture supernatant was extracted with a QIAamp Viral RNA mini (QIAGEN, Venlo, Netherlands) or MagMax Viral/Pathogen II Nucleic Acid Isolation kit (Thermo Fisher Scientific, Waltham, MA, USA) and quantified by real-time RT-PCR analysis with a one-step qRT-PCR kit (THUNDERBIRD Probe One-step qRT-PCR kit, TOYOBO, Osaka, Japan) using 5′-ACAGGTACGTTAATAGTTAATAGCGT-3′, ′-ATATTGCAGCAGTACGCACACA-3′, and 5′-FAM-ACACTAGCCATCCTTACTGCGCTTCG-TAMRA-3′ (E-set) [31].

### 4.5. Detection of Viral N Protein

The viral N protein was detected using a rabbit anti-SARS-CoV N antibody [32] as a primary antibody with AlexaFluor 568 anti-rabbit IgG or anti-rabbit IgG-HRP (Thermo Fisher, Waltham, MA, USA) as secondary antibodies together with DAPI to stain the nucleus by indirect immunofluorescence as described previously [33].

### 4.6. Quantification of Cell Viability

Cell viability was determined by MTT assay as previously reported [33].

### 4.7. Quantification of Transforming Growth Factor β (TGFβ) and Hedgehog (Hh) Activity

TGFβ activity was examined with NIH3T3 cells precultured with DMEM containing 0.1% bovine calf serum (BCS) overnight. NIH3T3 cells were pretreated with the compounds for 2 h and then stimulated with TGFβ1 (20 ng/mL) in the presence or absence of compounds. After 48 h, RNA was extracted from the cells and analyzed for quantifying the mRNA for a TGFβ target gene, connective tissue growth factor (CTGF), and Oaz1 for normalization. For examination of Hh activity, NIH3T3 cells pretreated with the compounds for 2 h were treated with conditioned medium from CAPAN-1 human pancreatic tumor cells that contain Shh in the absence or presence of the compounds. Cellular RNA was extracted and analyzed for the expression of a Hh target gene, Gli1, and normalized to Oaz1 expression.

### 4.8. Electron Microscopic Analysis

Cells were fixed with the buffer (2.5% glutaraldehyde, 2% paraformaldehyde, and 0.1 M phosphate buffer (pH 7.4)) for 1 h at room temperature followed by with 1% osmium tetroxide, stained in 1% uranyl acetate, dehydrated through a graded series of alcohols and embedded in Epon. Ultrathin sections were stained with uranyl acetate and lead citrate to observe with a transmission electron microscope (HT7700; Hitachi, Ltd., Tokyo, Japan).

### 4.9. Hepatitis C Virus (HCV) Replication Assay

HCV replication activity was measured using LucNeo#2 cells, carrying a subgenomic replicon RNA for an HCV NN strain (genotype-1b) and the luciferase gene driven by the HCV replication [30]. LucNeo#2 cells were treated with the compounds indicated in Figure 3C for 48 h, and the luciferase activity was measured with Luciferase Assay System kit (Promega, Madison, WI, USA). Sofosbuvir, a clinically used HCV polymerase inhibitor, was used as a positive control.

### 4.10. Hepatitis D Virus (HDV) Replication Assay

HDV was recovered from the culture supernatant of Huh7 cells transfected with the plasmids for HDV genome and for hepatitis B virus surface antigen [34]. HepG2-hNTCP-C4 cells were inoculated with HDV for 16 h and were further cultured for 6 days in the presence or absence of Oxy210 to detect intracellular HDV RNA [34]. Myrcludex-B (Myr-B), used as a positive control that inhibits HDV infection, was treated during the virus inoculation.

### 4.11. Pharmacokinetics of Oxy210 in Mice

We performed the pharmacokinetic analysis in mice by oral administration with Oxy210 as described previously [16].

## Figures and Tables

**Table 1:** The antiviral activities and cytotoxicities for all compounds.

Compounds	IC50 (μM)	IC90 (μM)	CC50 (μM)
Cholesterol	>30	>30	>30
4-beta-OHC	>30	>30	>30
7-KC	14.5	>30	>30
22(*S*)-OHC	>30	>30	>30
22(*R*)-OHC	13.2	>30	>30
24(*S*)-OHC	1.3	>30	>30
27-OHC	3.5	>30	>30
RDV	1.5	2.5	>10
Oxy133	>15	>15	>15
Oxy186	7.4	>15	>15
Oxy210	5.5	8.6	>15
Oxy232	5.4	7.8	>15

## Data Availability

The data presented in this study are available on request from corresponding author.

## Supplementary Materials

The following are available online at https://www.mdpi.com/1422-0067/22/6/3163/s1, Note, Table S1: Concentration of Oxy210 in plasma, liver, and lung in mice, Figure S1: Chemical structure of the natural oxysterols used in this study, Figure S2a: Proton spectrum of Oxy133, Figure S2b: Carbon spectrum of Oxy133, Figure S3a: Proton spectrum of Oxy186, Figure S3b: Carbon spectrum of Oxy186, Figure S4a: Proton spectrum of Oxy210, Figure S4b: Carbon spectrum of Oxy210, Figure S5a: Proton spectrum of Oxy232, Figure S5b: Carbon spectrum of Oxy232.

## Abbreviations

SARS-CoV-2	severe acute respiratory syndrome-related coronavirus 2
COVID-19	coronavirus disease 2019
PK	pharmacokinetics
DMVs	double-membrane vesicles
RDV	remdesivir
CPE	cytopathic effect
N protein	nucleocapsid protein
7-KC	7-ketocholesterol
4beta-OHC	4beta-hydroxycholesterol
22(S)-OHC	22(S)-hydroxycholesterol
22(R)-OHC	22(R)-hydroxycholesterol
24(S)-OHC	24(S)-hydroxycholesterol
27-OHC	27-hydroxycholesterol
IC_50_	50% maximal inhibitory concentration
IC_90_	90% maximal inhibitory concentration
CC_50_	50% maximal cytotoxic concentration
CLQ	chloroquine
HCV	hepatitis C virus
HDV	hepatitis D virus
CTGF	connective tissue growth factor
