# Supplementary material for: Identification of Anti-Severe Acute Respiratory Syndrome-Related Coronavirus 2 (SARS-CoV-2) Oxysterol Derivatives In Vitro
Source: Int J Mol Sci. 2021 Mar 19;22(6):3163. doi: 10.3390/ijms22063163 (PMC8003796; doi:10.3390/ijms22063163)

Supplementary Information

Identification of anti-severe acute respiratory syndrome-related coronavirus 2 (SARS-CoV-2) oxysterol derivatives in vitro

Hirofumi Ohashi[1,2], Feng Wang[3], Frank Stappenbeck[3], Kana Tsuchimoto[1], Chisa Kobayashi[1,2], Wakana Saso[1,4,5], Michiyo Kataoka[6], Masako Yamasaki[1,2], Kouji Kuramochi[2], Masamichi Muramatsu[1], Tadaki Suzuki[6], Camille Sureau[7], Makoto Takeda[8], Takaji Wakita[1], Farhad Parhami[3]*, and Koichi Watashi[1,2,9,10]*

[1]Department of Virology II, National Institute of Infectious Diseases, Tokyo 162-8640, Japan; hiro4@nih.go.jp (H.O.); kanatcmt@nih.go.jp (K.T.); k-chisa@nih.go.jp (C.K.); wsaso@nih.go.jp (W.S.); mayamasa@nih.go.jp (M.Y.); muramatsu@nih.go.jp (M.M.); wakita@nih.go.jp (T.W.)
[2]Department of Applied Biological Sciences, Tokyo University of Science, Noda 278-8510, Japan, kuramoch@rs.tus.ac.jp
[3]MAX BioPharma, Inc., 2870 Colorado Avenue, Santa Monica, CA 90404, USA; fwang@maxbiopharma.com (F.W.); fstappenbeck@maxbiopharma.com (F.S.)
[4]The Institute of Medical Science, The University of Tokyo, Tokyo 108-8639, Japan,
[5]AIDS Research Center, National Institute of Infectious Diseases, Tokyo 162-8640, Japan,
[6]Department of Pathology, National Institute of Infectious Diseases, Tokyo 162-8640, Japan, michiyo@nih.go.jp (M.K.); tksuzuki@nih.go.jp (T.S.)
[7]Laboratoire de Virologie Moléculaire, Institut National de la Transfusion Sanguine, Paris 75739, France, csureau@ints.fr
[8]Department of Virology III, National Institute of Infectious Diseases, Tokyo 208-0011, Japan, mtakeda@nih.go.jp
[9]Institute for Frontier Life and Medical Sciences, Kyoto University, Kyoto 606-8507, Japan,
[10]MIRAI, JST, Kawaguchi Center Building, 4-1-8 Honcho, Kawaguchi City, Saitama 332-0012, Japan,

* Correspondence: fparhami@maxbiopharma.com (F.P.); kwatashi@nih.go.jp (K.W.); Tel.: +1-424-581-6025 (F.P.); +81-3-5285-1111 (K.W.)

Received: date; Accepted: date; Published: date

Supplementary Note

Supplementary Table S1

Supplementary Figure S1-5

Supplementary Notes

Synthesis and Molecular Characterization of semi-synthetic oxysterol derivatives Oxy133, Oxy186, Oxy210 and Oxy232:

Materials were obtained from commercial suppliers and were used without further purification. Air or moisture sensitive reactions were conducted under an argon atmosphere using oven-dried glassware and standard syringe/septa techniques. The reactions were monitored on silica gel TLC plates under UV light (254 nm) followed by visualization with Hanessian's staining solution. Chromatographic purifications were performed using a Teledyne ISCO CombiFlash Rf automated chromatography system. NMR spectra were measured in CDCl3. The data are reported as follows in ppm from an internal standard (TMS, 0.0 ppm): chemical shift (multiplicity, integration, coupling constant in Hz.). The carbon and proton spectra for Oxy133, Oxy186, Oxy210, and Oxy232 are shown in Supplementary Figure S2-5.

__Synthesis of Oxy133:__

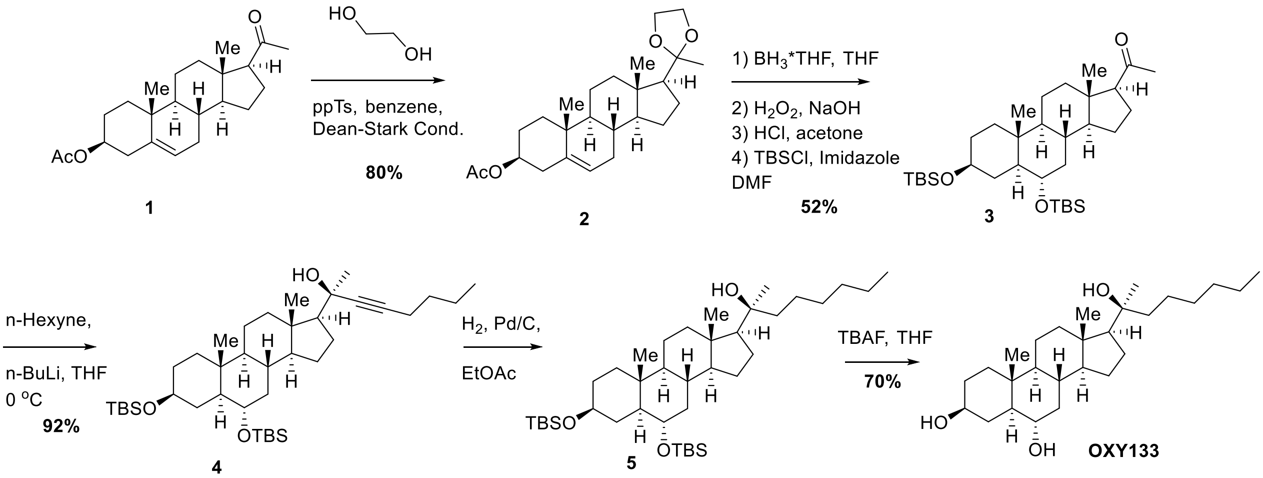

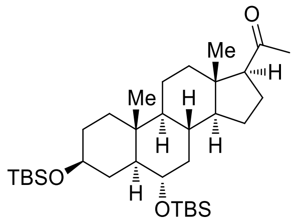

1-((3S,5S,6S,8R,9S,10R,13S,14S,17S)-3,6-bis((tert-butyldimethylsilyl)oxy)-10,13-dimethylhexadecahydro-1H-cyclopenta[a]phenanthren-17-yl)ethanone (**3**)

Prepared according to a published patent procedure.[1]

^{1}H NMR ($CDCl_3$, 400 MHZ) δ: 3.47 (1H, dddd, J = 11.0, 11.0, 4.8, 4,8 Hz), 3.36 (1H, ddd, J = 10.4, 10.4, 4.4 Hz), 2.53 (1H, d, J = 8.8, 8.8 Hz), 2.20-2.14 (1H, m), 2.10 (3H, s), 2.01-1.97 (1H, m), 1.88-1.82 (1H, m), 1.73-0.89 (17H, m), 0.88, 18H, s), 0.79 (3H, s), 0.59 (3H, s), 0.043 (3H, s), 0.04 (3H, s), 0.03 (3H, s), 0.02 (3H, s). ^{13}C NMR ($CDCl_3$, 100 MHZ) δ: 209.5, 72.2, 70.1, 63.7, 56.4, 53.7, 51.8, 44.2, 41.9, 38.9, 37.6, 36.3, 34.3, 33.2, 31.7, 31.5, 25.94, 25.92, 24.4, 22.7, 21.1, 18.3, 18.1, 13.5, 13.4, -4.1, -4.6, -4.7.

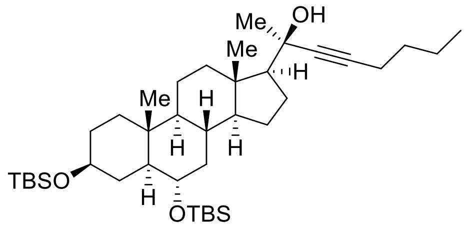

(R)-2-((3S,5S,6S,8R,9S,10R,13S,14S,17S)-3,6-bis((tert-butyldimethylsilyl)oxy)-10,13-dimethylhexadecahydro-1H-cyclopenta[a]phenanthren-17-yl)oct-3-yn-2-ol (**4**)

To a cold (0 oC) solution of n-hexyne (1.5 mL, 12 mmol) in THF (6 mL) was added a 1.6 M solution of n-BuLi in hexanes (3.75 mL). The resulting solution was stirred for 30 min until a solution of 1-((3S,5S,6S,8R,9S,10R,13S,14S,17S)-3,6-bis((tert-butyldimethylsilyl)oxy) -10,13-dimethylhexadecahydro-1H-cyclopenta[a]phenanthren-17-yl)ethanone (**3**), (1.27 g, 2.2 mmol) in THF (10 mL) was added via cannula. The mixture was warmed to room temperature over 3 h and diluted with water (40 mL) and the crude product was isolated by ethyl acetate extraction (3 x 30 mL). The combined organic layers were washed with brine and dried over Na2SO4.

[1] Parhami, F.; Jung, M.E.; Nguyen, K.; Yoo, D.; Kim, W. WO 2009/07386, pp.52.

Concentration gave an oily product which was purified on silica gel (hexane, EtOAc, gradient). There was 1.30 g of (R)-2-((3S,5S,6S,8R,9S,10R,13S,14S,17S)- 3,6-bis((tert-butyldimethylsilyl)oxy)-10,13-dimethylhexadecahydro-1H-cyclopenta[a]phenanthren-17-yl)oct-3-yn-2-ol (**4**) (92%).

^{1}H NMR ($CDCl_3$, 300 MHZ) δ: 3.50 (1H, ddd, J = 15.9, 11.0, 4.8 Hz), 3.36 (1H, dt, J = 10.6, 4.3 Hz), 2.18 (1H, t, J = 6.9 Hz), 2.10 (1H, m), 1.91-1.62 (4H, m), 1.53-1.31 (2H, m, 3H, s), 1.31-0.93 (22 H, m), 0.93 (3H, s), 0.92 (3H, m), 0.90 (18H, s), 0.88 (3H, s), 0.61 (1H, m), 0.04 (6H, s), 0.03 (6H, s). ^{13}C NMR ($CDCl_3$, 75 MHZ) δ: 85.9, 83.9, 72.4, 71.4, 70.3, 60.5, 55.8, 53.8, 51.8, 43.5, 36.3, 33.7, 33.0, 30.7, 25.9, 22.0, 18.4, 18.3, 18.1, 13.6, 13.5, -4.7, -4.7.

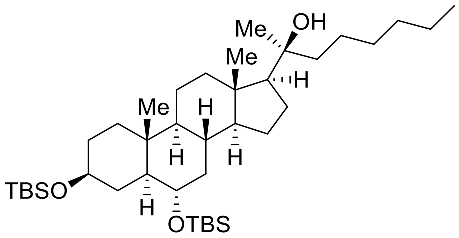

(S)-2-((3S,5S,6S,8R,9S,10R,13S,14S,17S)-3,6-bis((tert-butyldimethylsilyl)oxy)-10,13-dimethylhexadecahydro-1H-cyclopenta[a]phenanthren-17-yl)octan-2-ol (**5**)

(R)-2-((3S,5S,6S,8R,9S,10R,13S,14S,17S)-3,6-bis((tert-butyldimethylsilyl)oxy)-10,13-dimethylhexadecahydro-1H-cyclopenta[a]phenanthren-17-yl)oct-3-yn-2-ol (**4**), (1.3 g, 2.0 mmol) was dissolved in EtOAc (5 mL), MeOH (5 mL) and Pd/C (10%, 0.1 g) was added to the solution. The mixture was degassed repeatedly under vacuum and then exposed to hydrogen gas under atmospheric pressure (balloon). After 18 h at room temperature, the mixture was diluted with EtOAc (20 mL) and filtered over Celite to remove the catalyst. The filter washed with EtOAc and the combined filtrates evaporated. There was 1.3 g of reduced product which was used without further purification.

^{1}H NMR ($CDCl_3$, 300 MHZ) δ: 3.50 (1H, ddd, J = 15.9, 11.0, 4.8 Hz), 3.36 (1H, dt, J = 10.6, 4.3 Hz), 2.1-1.95 (2H, m), 1.75-1.35 (10H, m), 1.32-1.29 (10H, m, 3H, s), 0.91-1.21 (10H, m), 0.89 (18H, s), 0.82 (3H, s), 0.79 (3H, s), 0.63 (3H, m), 0.04 (6H, s), 0.03 (6H, s) ^{13}C NMR ($CDCl_3$, 75 MHZ) δ: 75.2, 72.3, 57.6, 56.4, 53.8, 51.8, 42.9, 37.6, 36.3, 33.7, 31.9, 30.0, 25.9, 22.6, 18.3, 18.1, 14.1, 13.8, 13.5, -4.6, -4.7.

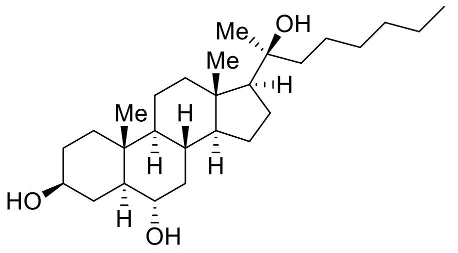

(3S,5S,6S,8R,9S,10R,13S,14S,17S)-17-((S)-2-hydroxyoctan-2-yl)-10,13-dimethylhexadecahydro-1H-cyclopenta[a]phenanthrene-3,6-diol (**Oxy133**).

A 1 M solution of TBAF in THF (8 mL, 8 mmol, 4 equiv) was directly added to (S)-2-((3S,5S,6S,8R,9S,10R,13S,14S,17S)-3,6-bis((tert-butyldimethylsilyl)oxy)-10,13-dimethylhexadecahydro-1H-cyclopenta[a]phenanthren-17-yl)octan-2-ol (**5**), (1.3 g, 2.0 mmol, 1.0 equiv) and the resulting solution was diluted with THF (1 mL) and stirred at room temperature for 72 h. The mixture was then diluted with water (50 mL) and extracted repeatedly with EtOAc (4 x 40 mL). The combined organic layers were washed with brine, dried over Na2SO4 and the solvent evaporated. Purification of the crude product by silica gel chromatography (hexane, EtOAc, gradient, then 10% MeOH in EtOAc) afforded a white solid (0.6 g, 70%) which was subjected to trituration in aqueous acetone (acetone, water, 3:1).

^{1}H NMR ($CDCl_3$, 300 MHZ) δ: 3.50 (1H, ddd, J = 15.9, 11.0, 4.8 Hz), 3.36 (1H, dt, J = 10.6, 4.3 Hz), 2.19 (1H, m), 2.10-1.90 (3 H, m), 1.85-1.60 (7 H, m), 1.55-1.38 (7H, m), 1.25 (11H, brs), 1.20-0.95 (4 H, m), 0.90 (3H, m), 0.86 (3H, s), 0.80 (3H, s) 0.62 (1H, m). ^{13}C NMR ($CDCl_3$, 75 MHZ) δ: 75.1, 71.1, 69.3, 57.5, 56.2, 53.6, 51.6, 44.0, 42.8, 41.4, 40.1, 37.2, 36.2, 33.5, 32.1, 31.8, 30.9, 29.9, 26.3, 24.2, 23.6, 22.5, 22.2, 20.9, 14.0, 13.6, 13.3. MS: M+H = 420.36. HRMS (ESI) m/z [M- 2(H2O) + H]+ calcd for C27H44OH: 385.3470, found 385.3478.

__**Synthesis of Oxy186:**__

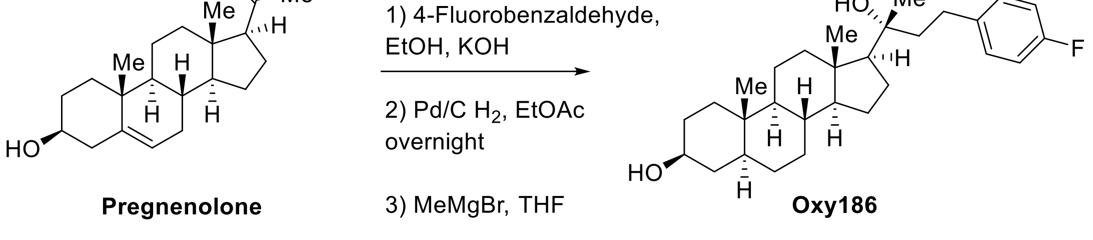

Oxy186 was prepared in three synthetic steps as shown above. Briefly, pregnenolone was condensed with 4-fluorobenzaldehyde to the enone which was reduced along with the C-5,6 double bond by hydrogenation using palladium on carbon (Pd/C) as a catalyst. The resulting fully saturated ketone was reacted with methylmagnesium bromide to afford Oxy186. The crude product was purified by chromatography on silica. (3S,5S,8R,9S,10S,13S,14S,17S)- 17-((R)-4-(4-fluorophenyl)-2-hydroxybutan-2-yl)-10,13-dimethylhexadecahydro-1H-cyclopenta[a]phenanthren-3-ol (**Oxy186**):

^{1}H NMR ($CDCl_3$, 400 MHZ) δ: 7.14- 7.11 (2H, m), 6.97 (2H, dd, J = 8.8, 8.8 Hz), 3.54 (1H, dddd, J = 0.9,10.9, 5.5, 5.5 Hz), 2.73- 2.64 (2H, m), 2.32-0.63 (15H, m), 1.21 (3H, s), 0.80 (3H, s), 0.76 (3H, s). ^{13}C NMR ($CDCl_3$, 100 MHZ) δ: 161.2 (d, J = 242 Hz), 138.2 (d, J = 3.1), 129.6 (d, J = 20 Hz), 115.1 (d, J = 20 Hz), 75.7, 71.3, 58.8, 56.7, 54.3, 44.9, 44.7, 43.3, 40.6, 38.15, 37.0, 35.5, 34.9, 32.0, 31.5, 29.6, 28.7, 23.8, 26.8, 23.7, 23.3, 21.1, 14.0, 12.3.

Synthesis of Oxy210:

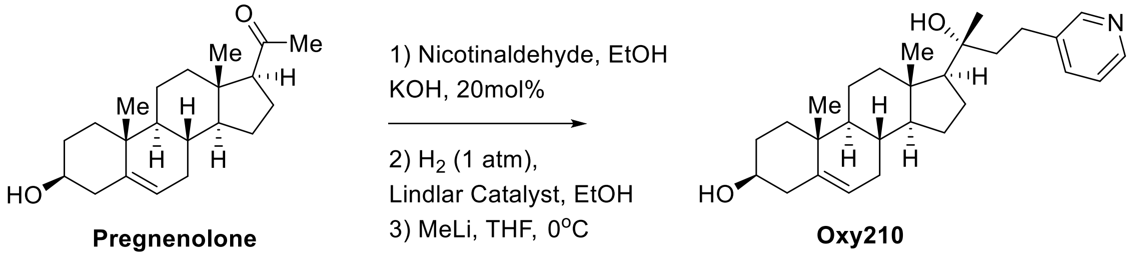

Oxy210 was prepared in three synthetic steps as shown above. Pregnenolone was condensed with nicotinaldehyde to the enone which was reduced via hydrogenation using Lindlar's catalyst. The saturated ketone was reacted with methyllithium to afford Oxy210. The crude product was purified by chromatography on silica. (3S,8S,9S,10R,13S,14S,17S)-17- ((R)-2-hydroxy-4-(pyridin-3-yl)butan-2-yl)-10,13-dimethyl-2,3,4,7,8,9,10,11,12,13,14,15,16,17-tetradecahydro-1H-cyclopenta[a]phenanthren-3-ol (**Oxy210**):

^{1}H NMR (400 MHz, $CDCl_3$) δ 8.45 (1H, d, J= 1Hz), 8.42 (1H, dd, J= 5, 2 Hz), 7.53-7.48 (1H, m), 7.23-7.18 (1H, m), 5.35-5.31 (1H, m), 3.56-3.45 (1H, m), 2.79-2.63 (2H, m), 2.33-2.17 (2H, m), 2.05 (1H, m), 2.01-1.26 (16 H, m), 1.23 (3H, s), 1.18-0.89 (3H, m), 0.98 (3H, s), 0.87 (3H, s); ^{13}C NMR (100 MHz, $CDCl_3$) δ 149.7, 147.1, 140.8, 138.1, 135.8, 128.6, 123.4, 121.4, 75.5, 71.6, 58.7, 56.9, 50.0, 44.1, 42.9, 42.3, 40.3, 37.2, 36.5, 31.7, 31.6, 31.3, 27.5, 26.7, 23.7, 23.2, 20.9, 19.3, 13.7.

__Synthesis of Oxy232:__

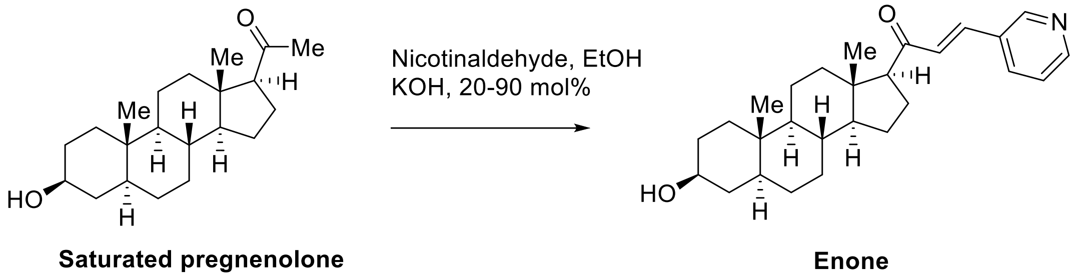

Step 1: **Saturated pregnenolone** (1.2 g, 3.8 mmol) was suspended in ethanol (20 mL) at room temperature. A potassium hydroxide solution (4M, 0.2 mL, 0.2 equivalents) was added to the reaction mixture followed by addition of nicotinaldehyde (0.7 g, 6.5 mmol). The resulting mixture was stirred at room temperature for 24 hours. Upon completion of the reaction (TLC analysis), water (50 mL) was added to the reaction mixture to precipitate the product. The crude solid product was isolated using vacuum filtration, washed with water (2 x 20 mL) and then air dried. There was obtained 1.56 g (> 95 %) of **enone** product.

^{1}H NMR (400 MHz, $CDCl_3$) δ 8.75 (1H, d, J= 2 Hz), 8.57 (1H, dd, J= 5, 2Hz), 7.86-7.81 (1H, m), 7.50 (1H, d, J= 17Hz), 7.31 (1H, dd, J= 8, 4 Hz), 6.81 (1H, d, J= 17 Hz), 3.56-3.40 (1H, m), 2.83 (1H, dd, J= 9, 9 Hz), 2.39-2.17 (3H, m), 2.06-1.95 (3H, m), 1.87-1.01 (14H, m), 0.81 (3H, s), 0.62 (3H, s).

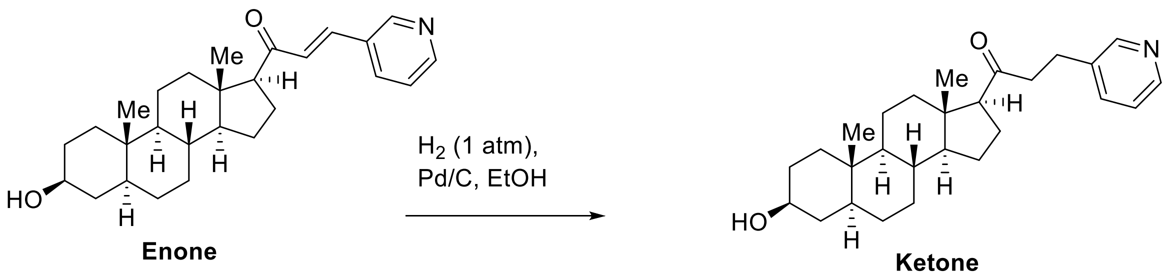

Step 2: The enone (1.5 g, 3.8 mmol) was suspended in ethanol (25 mL) and ethyl acetate (5ml) at room temperature and palladium on carbon catalyst (0.15 g) was added to the mixture. The atmosphere in the reaction flask was purged three times with hydrogen gas using a balloon. The reaction mixture was then stirred at room temperature under a hydrogen atmosphere. After 2

days, the mixture was filtered over celite and concentrated in vacuo. The crude product mixture was purified via automated chromatography (ISCO) running an hexanes/ethyl acetate gradient (0-100%) to yield pure **ketone** product (1.0 g, 66%).

^{1}H NMR (400 MHz, $CDCl_3$) δ 8.42 (1H, d, J = 2Hz), 8.40 (1H, dd, J = 5,1 Hz), 7.52-7.47 (1H, m), 7.17 (1H, dd, J= 8, 5 Hz), 3.54-3.44 (1H, m), 2.92-2.91 (2H, m), 2.72-2.64 (2H, m), 2.45, (1H, dd, J= 9, 9 Hz), 2.35-1.00 (17H, m), 0.96 (3H, s), 0.52 (3H, s); ^{13}C NMR (125 MHz, $CDCl_3$) δ 209.9, 149.8, 147.5, 136.9, 136.2 123.3, 71.2, 63.2, 56.8, 54.2, 45.3, 44.8, 44.6, 39.2, 38.1, 37.0, 35.5, 32.0 31.5, 28.6, 26.8 24.4, 23.0, 21.0,13.3, 12.3.

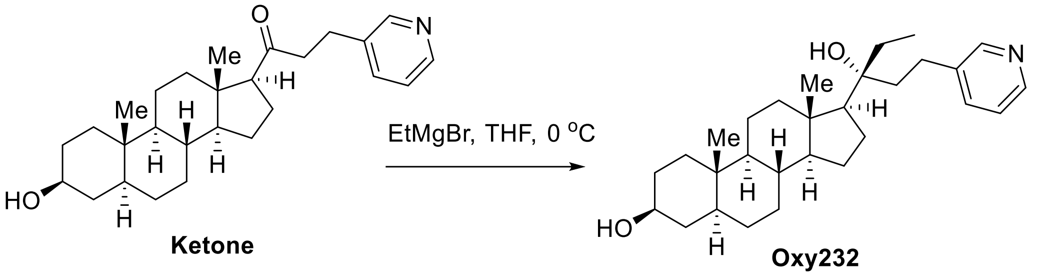

Step 3: The ketone (0.41 g, 1 mmol) was dissolved in dry tetrahydrofuran (5 mL) at room temperature and cooled to 0 oC under N2-atmosphere. A solution of ethyl magnesium bromide (3 M in ether, 2 mL, 6 mmol) was added dropwise to the reaction mixture at 0 oC. The reaction mixture was then stirred at 0 oC for 1 hour until the starting material was mostly consumed (TLC analysis). Then the reaction was carefully quenched with a small volume (~1 mL) of methanol and the mixture further diluted with saturated ammonium chloride solution (20 mL) and dichloromethane (20 mL). The layers were separated, and the aqueous layer extracted with dichloromethane (2 x 30 mL). The combined organic layers were dried over sodium sulfate, filtered and concentrated in vacuo. The crude mixture was purified via automated chromatography (ISCO) running an ethyl acetate/methanol gradient (0-10%) to yield pure (3S,5S,8R,9S,10S,13S,14S,17S)-17-((R)-3-hydroxy-1-(pyridin-3-yl)pentan-3-yl)-10,13-dimethylhexadecahydro-1H-cyclopenta[a]phenanthren-3-ol (**Oxy232**) (0.35 g, 80 %).

^{1}H NMR (400 MHz, $CDCl_3$) δ 8.42 (1H, d, J= 1Hz), 8.38 (1H, dd, J= 5, 2 Hz), 7.53-7.48 (1H, m), 7.23-7.18 (1H, m), 3.54 (1H, m), 2.69-2.63 (2H, m), 2.33-2.17 (2H, m), 2.05 (1H, m), 2.01-1.26 (17 H, m), 1.22 (2H, m), 1.18-0.60 (6 H, m), 0.85 (3H, s), 0.77 (3H, s); ^{13}C NMR (100 MHz, $CDCl_3$) δ 149.1, 146.6, 138.5, 136.4, 123.6, 77.3, 71.2, 56.8, 55.3, 54.3, 44.9, 43.0, 40.7, 38.8, 38.2, 37.0, 35.4, 34.8, 32.0, 31.5, 31.0, 28.7, 27.3, 23.6, 22.3, 21.1,13.8, 12.3, 8.4.

Supplementary Table S1.

(**A**) Plasma concentrations of Oxy210

Time (h)	Sample concentration (ng/mL)					Mean ± SD
24	26	267	8	12	83	79 ± 109
48	1039	652	1218	764	622	859 ± 259
96	1157	771	4471	6050	959	2682 ± 2423

(**B**) Liver concentrations of Oxy210

Time (h)	Sample concentration (ng/g)					Mean ± SD
96	5227	3470	7302	13291	5055	6869 ±1717

(**C**) Lung concentrations of Oxy210

Time (h)	Sample concentration (ng/g)					Mean ± SD
96	1688	1109	6083	10524	1282	4137 ± 1843

Supplementary Table S1. **Concentration of Oxy210 in plasma, liver, and lung in mice.** Oxy210 mixed in regular Chow food at 4 mg/g of chow was fed to male C57BL/6 mice ad libitum. (**A**) Blood was drawn after 24 h, 48 h and 96h to analyze the plasma concentration of Oxy210. After 96 h, terminal liver (**B**) and lung (**C**) tissue samples were analyzed for Oxy210 concentrations. The data show the concentrations of Oxy210 in each tissue in five mice and its mean values and SD.

Supplementary Figure S1.

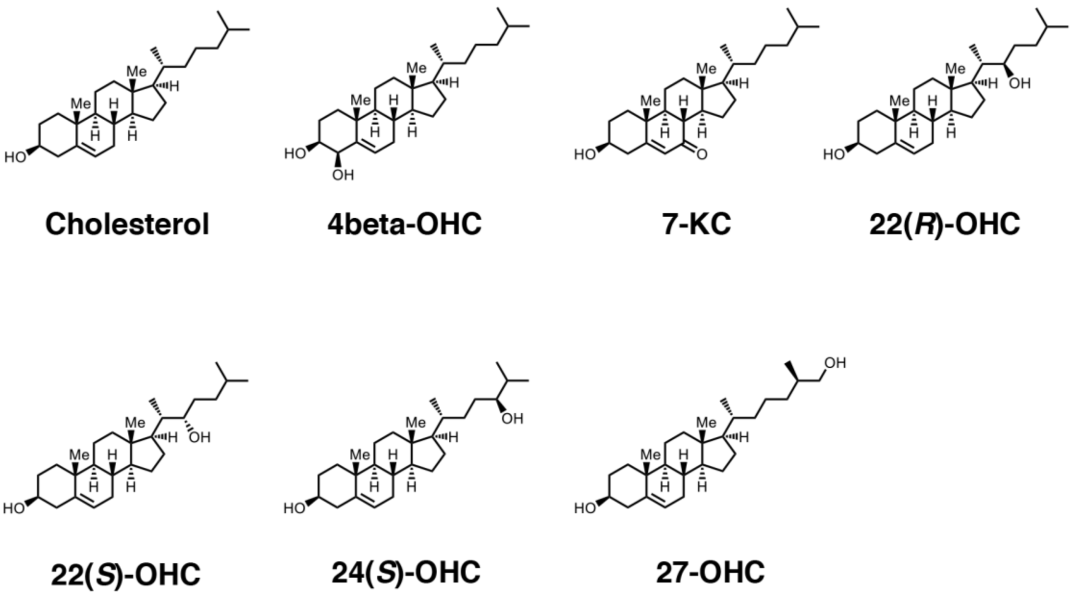

Supplementary Figure S1.
Chemical structure of the natural oxysterols used in this study.

Supplementary Figure S2a. Proton spectrum of Oxy133

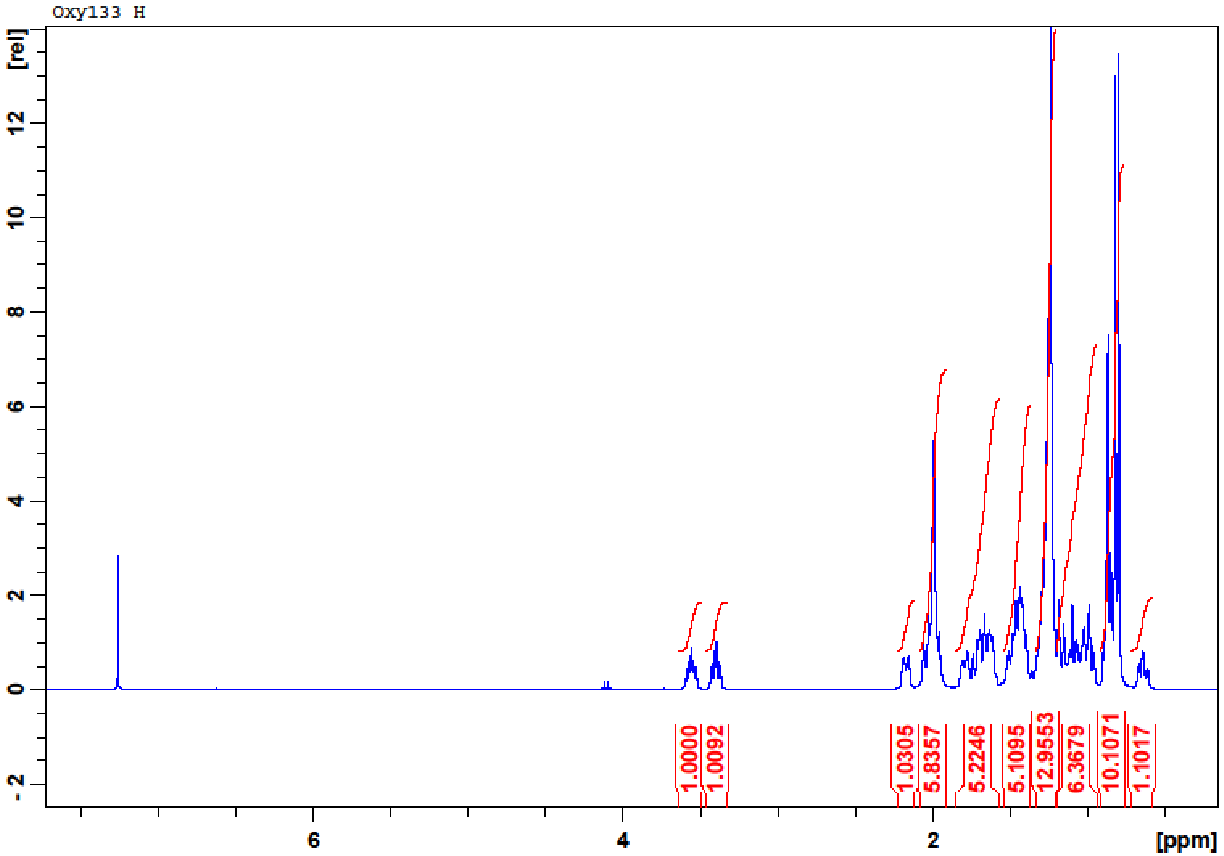

Supplementary Figure S2b. Carbon spectrum of Oxy133

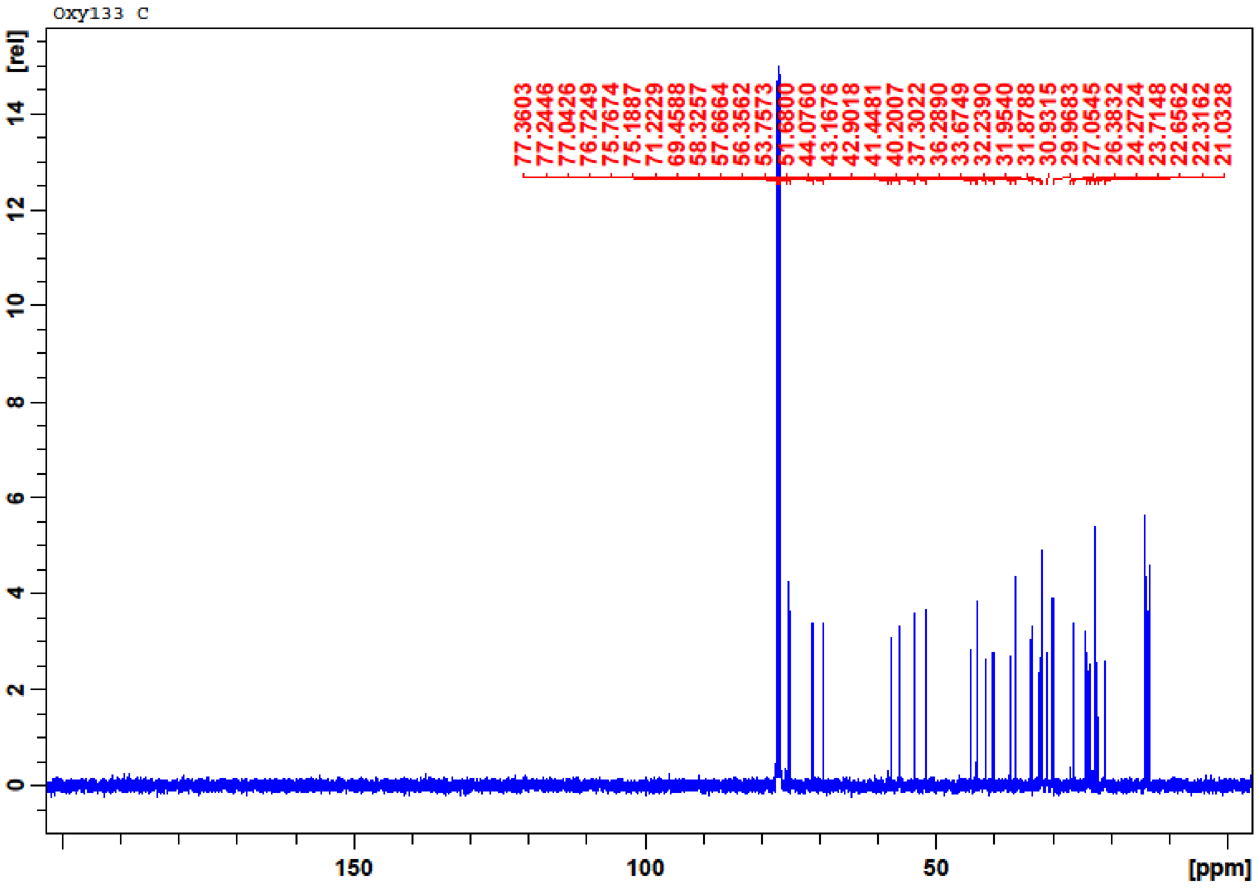

Supplementary Figure S3a. Proton spectrum of Oxy186

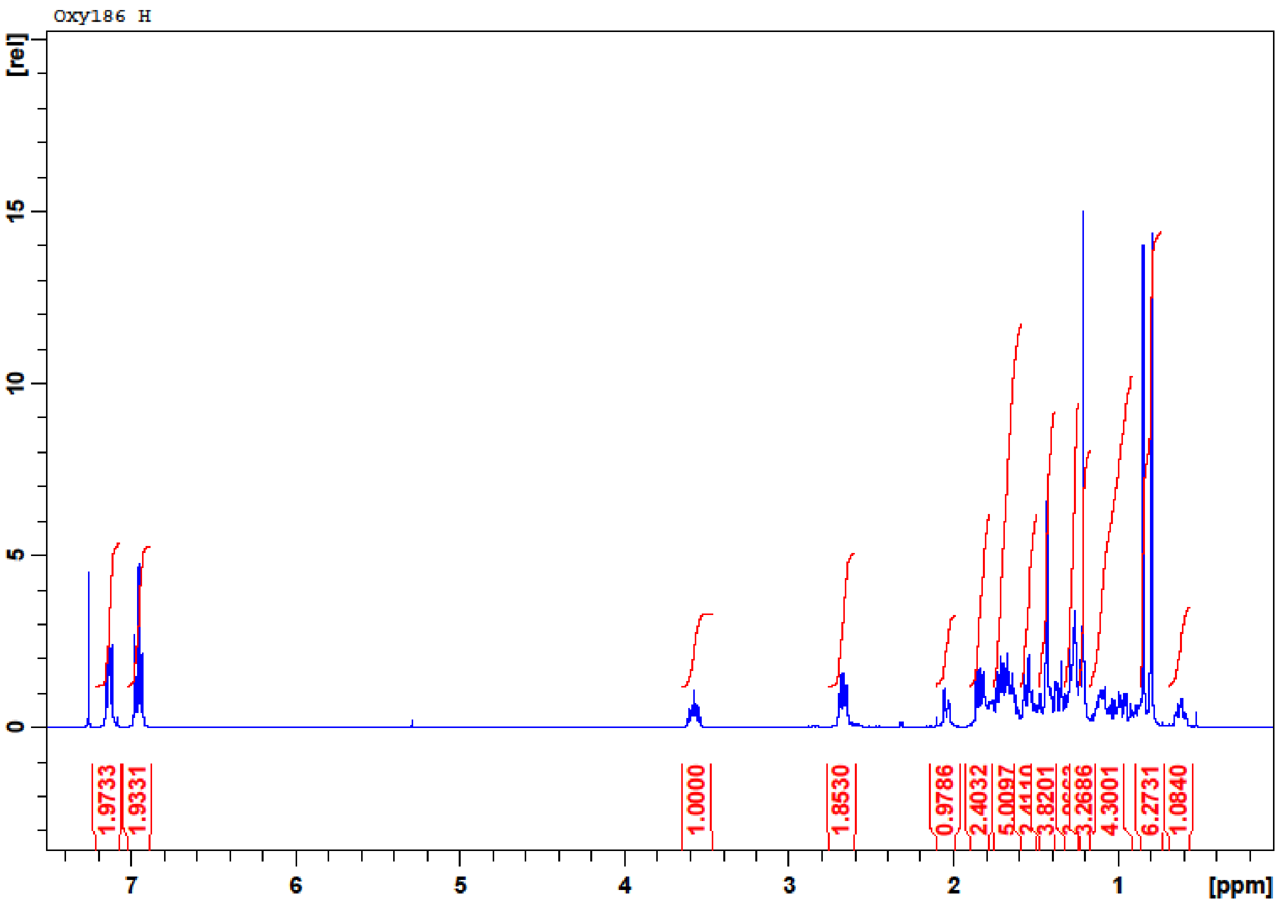

Supplementary Figure S3b. Carbon spectrum of Oxy186

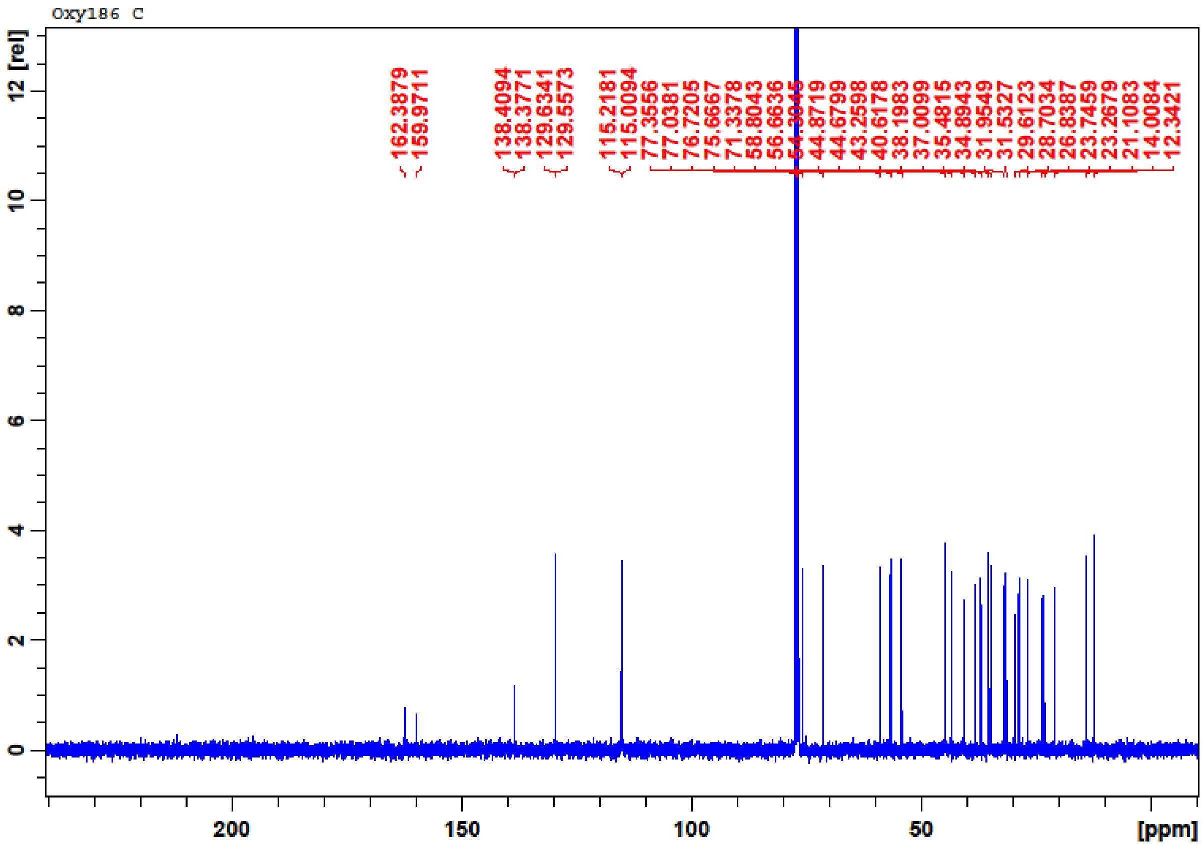

Supplementary Figure S4a. Proton spectrum of Oxy210

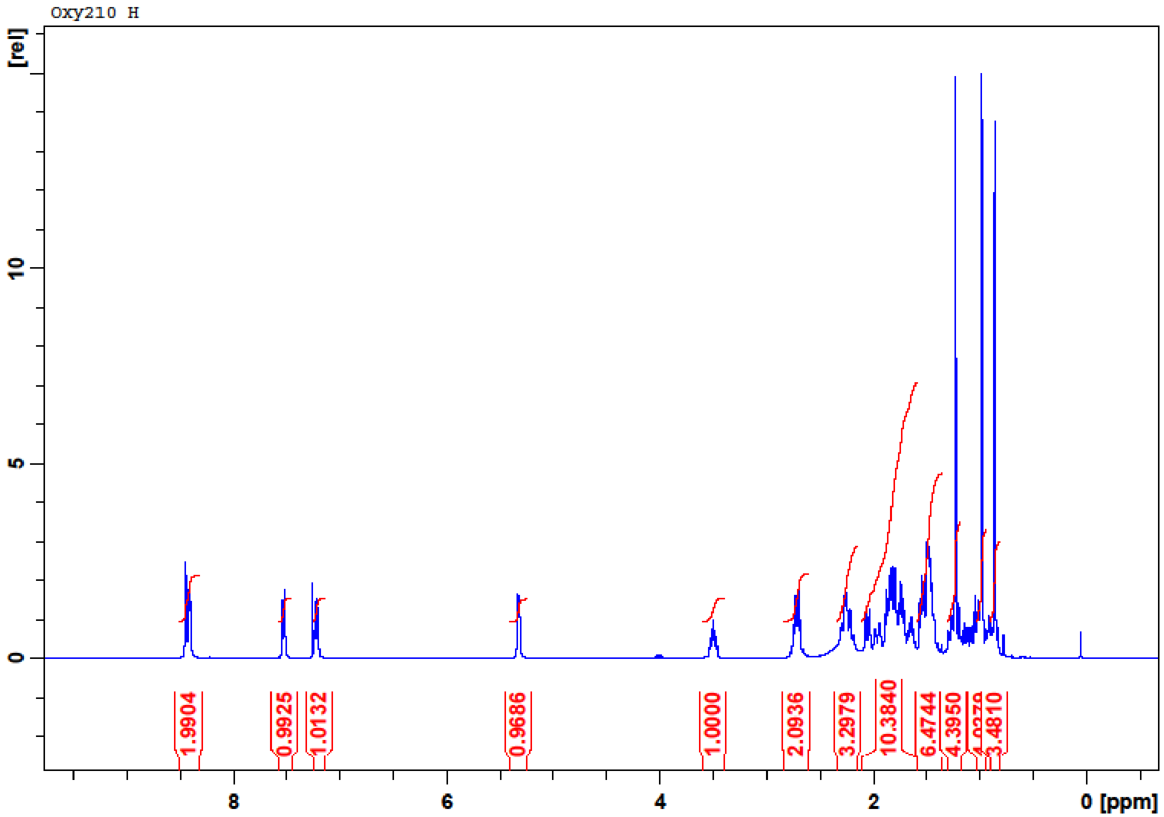

Supplementary Figure S4b. Carbon spectrum of Oxy210

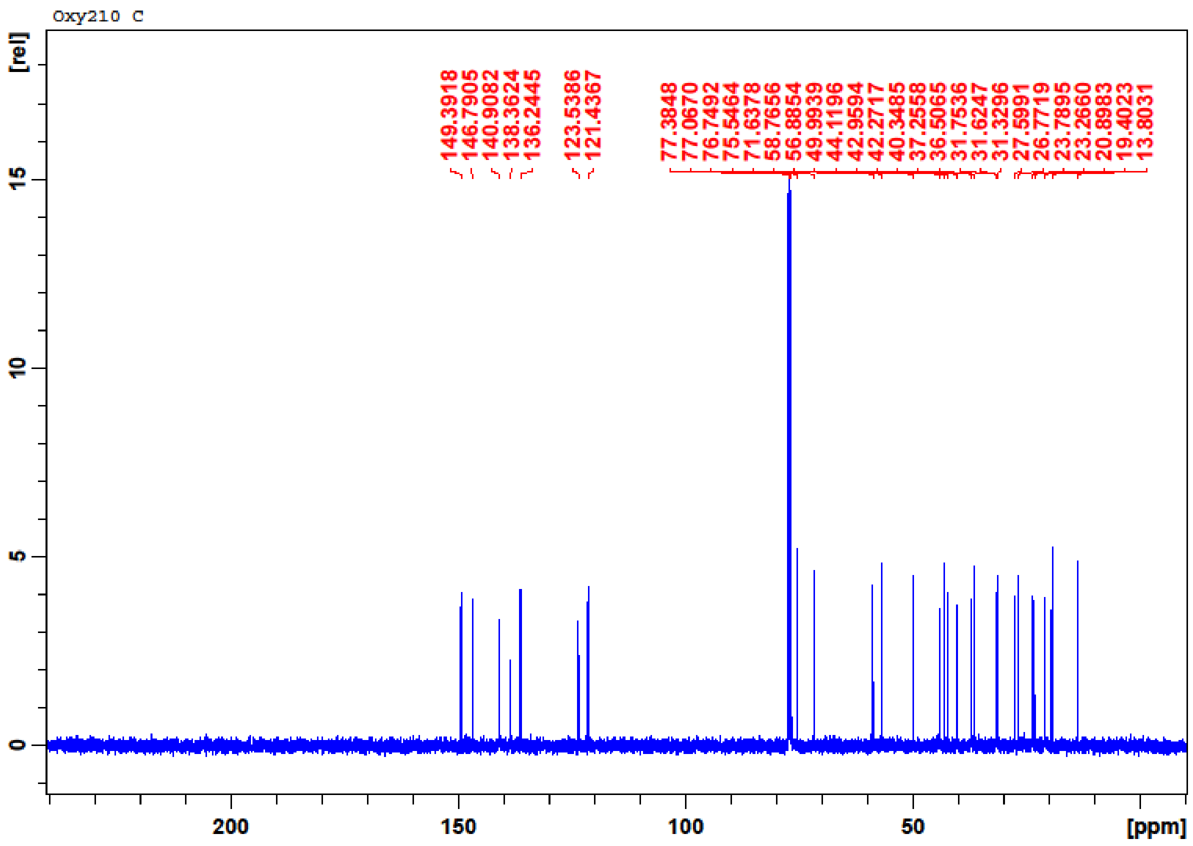

Supplementary Figure S5a. Proton spectrum of Oxy232

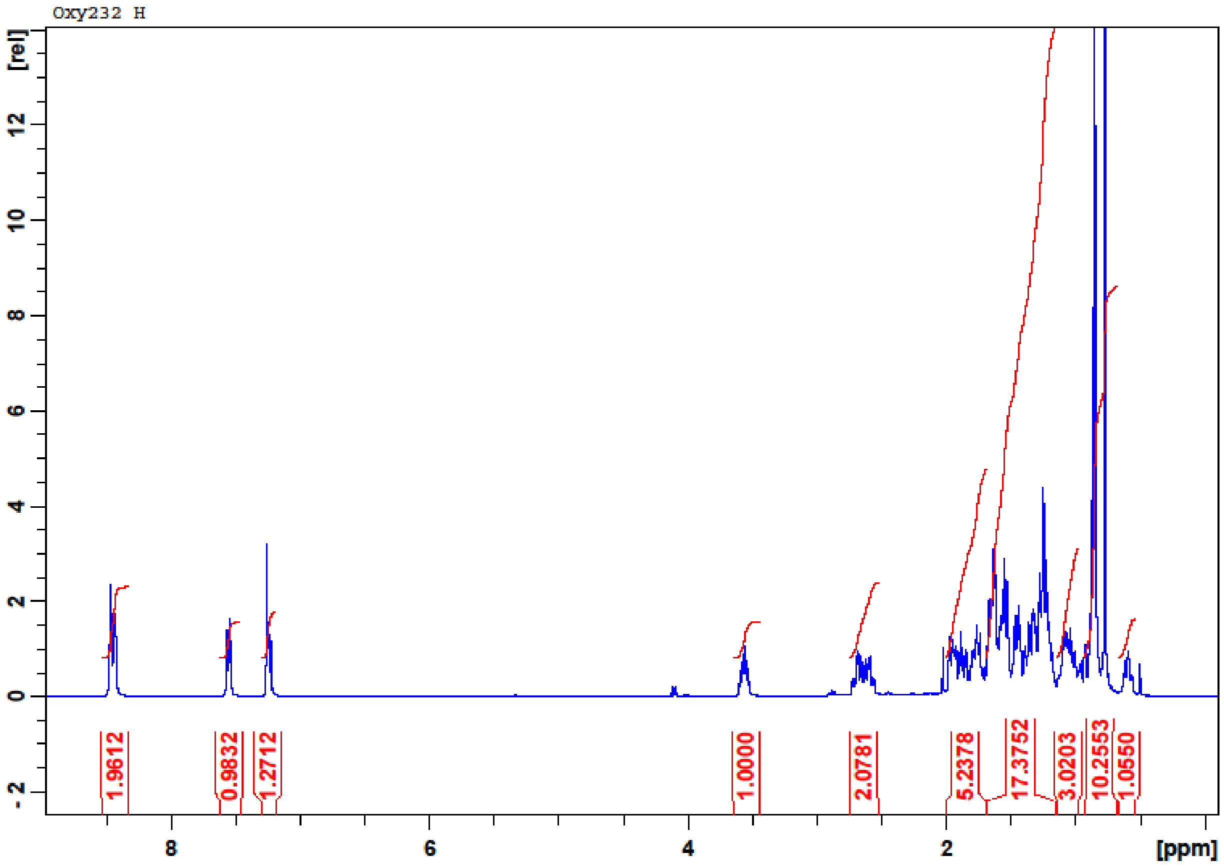

Supplementary Figure S5b. Carbon spectrum of Oxy232

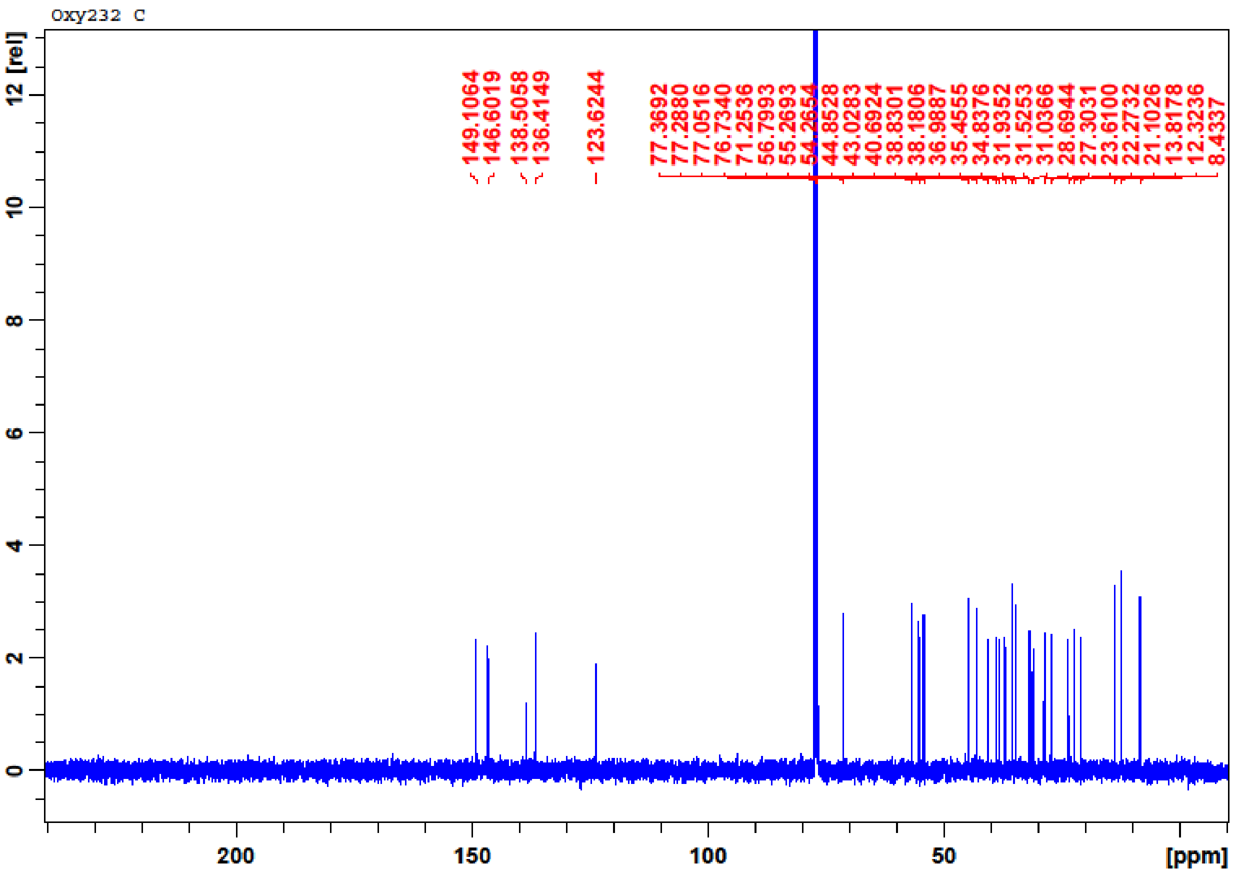

Supplement: Supplementary file 1 [file ijms-22-03163-s001.pdf]
